# Nanocarbon/Poly(Lactic) Acid for 3D Printing: Effect of Fillers Content on Electromagnetic and Thermal Properties

**DOI:** 10.3390/ma12152369

**Published:** 2019-07-25

**Authors:** Giovanni Spinelli, Patrizia Lamberti, Vincenzo Tucci, Rumiana Kotsilkova, Evgeni Ivanov, Dzhihan Menseidov, Carlo Naddeo, Vittorio Romano, Liberata Guadagno, Renata Adami, Darya Meisak, Dzmitry Bychanok, Polina Kuzhir

**Affiliations:** 1 Department of Information and Electrical Engineering and Applied Mathematics, University of Salerno, Via Giovanni Paolo II, 84084 Fisciano (SA), Italy; 2 Institute of Mechanics, Bulgarian Academy of Sciences, Acad. G. Bonchev Str., Block 4, 1113 Sofia, Bulgaria; 3 Research and Development of Nanomaterials and Nanotechnologies (NanoTech Lab Ltd.), Acad. G. Bonchev Str. Block 1, 1113 Sofia, Bulgaria; 4 Department of Industrial Engineering, University of Salerno, Via Giovanni Paolo II, 84084 Fisciano (SA), Italy; 5 Institute for Nuclear Problems of Belarusian State University, Bobruiskaya 11, 220030 Minsk, Belarus; 6 Radioelectronics Department, Faculty of Radiophysics, Tomsk State University, 36 Lenin Prospekt, Tomsk 634050, Russia

**Keywords:** 3D prototyping, 3D filaments, additive manufacturing, multi-wall carbon nanotubes, graphene platelets, PLA, thermal, electric and electromagnetic properties

## Abstract

Electromagnetic and thermal properties of a non-conventional polymer nanocomposite based on thermoplastic Polylactic acid (PLA, Ingeo™) filled, in different weight percentage, with multi-walled carbon nanotubes (MWCNTs), graphene nanoplatelets (GNPs), as well as a mixture of both fillers (MWCNTs/GNPs), are analyzed. The combination of notable electrical, thermal, and electromagnetic (EM) properties of the carbon fillers, in concentrations above the percolation threshold, together with the good processability of the PLA matrix gives rise to innovative filaments for 3D printing. In particular, the shielding efficiency (SE) in the frequency range 26–37 GHz of samples increases from 0.20 dB of unfilled PLA up to 13.4 dB for composites containing MWCNTs and GNPs, corresponding to 4% and 95% of SE, respectively. The thermal conductivity of the PLA loaded with 12 wt % of GNPs is 263% higher than that of the unfilled polymer, whereas an improvement of about 99% and 190% is detected for the PLA matrix loaded with MWCNTs and both fillers, respectively. The EM and thermal characterization is combined with a morphological investigation allowing us to correlate the dispersion states of the fillers within the polymer matrix with the observed EM and thermal properties. The EM and thermal characteristics exhibited by the nanocomposites make them suitable for packaging applications of electronic devices with electromagnetic interference (EMI) shielding and thermal dissipation features.

## 1. Introduction

In the last decade, additive manufacturing (i.e., AM), also known as 3D printing (i.e., 3DP), has drawn strong attention from academic as well as industrial fields since it allows easy, quick, and thorough production of three-dimensional complicated structures with a wide range of sizes, shapes, and functional properties [1]. This technology favors rapid prototyping since it does not require any molds as for conventionally machined parts, thus, offering different advantages in terms of ease of use, mass customization, tangible product testing, reliability, and cost-effectiveness [2].

Among the different additive manufacturing approaches, such as selective laser sintering, inkjet 3D printing, and solvent-cast 3DP (SC3DP), the fused deposition modeling (FDM) has pervaded most of the different fields given its simplicity [3,4]. In fact, the feedstock is generally a thermoplastic polymer filament which, after being heated above its glass transition temperature (i.e., *Tg*), is extruded by means of a PC-controlled moving nozzle. This process allows to form, layer by layer, the desired 3D structure [5].

However, a serious limitation of this technology is the possibility to print electrically conductive parts, since the choice of matrices is still limited to insulating ones (in general polyester, acrylonitrile butadiene styrene-ABS, nylon, polyvinyl alcohol-PVA, polycarbonate, and poly(lactic) acid-PLA). More recently, this issue is being addressed with the introduction of nanotechnology in the field of additive manufacturing the inclusion of suitable nanofillers into printable resins [6]. This combination between additive manufacturing and nanotechnology paves the way for the development of 3D printable materials exhibiting multifunctionality and customized properties, thus leading to an expansion of AM application areas [7,8].

Along this stream, the possibility of using poly(vinyl alcohol) PVA filled with ultra-dispersed diamonds as new materials for 3D printing is investigated in [9] where the main factors conditioning the quality of the final printed parts together with material design and preparation are discussed. The electromagnetic interference (EMI) shielding of various 3D printed polymeric composite structures based on commercially available filaments is analyzed in the so-called C-band of the electromagnetic spectrum (3.5–7.0 GHz) since it is generally exploited for long-distance radio telecommunications [10]. Moreover, given the possibility to print electrically conductive parts, in recent years, 3D printing technology has been adopted for the design of affordable and versatile antennas [11,12], also made with combined technologies [13], or for the realization of flexible and stretchable microelectrodes for interconnections in electronic and optoelectronic devices [14]. Additive manufacturing has also been widely adopted in the aerospace sector for the rapid prototyping and subsequent production of complex components otherwise requiring particular technological solutions and long design times [15]. The EM properties of poly(lactic) acid (PLA) reinforced with graphite nanoplatelets (GNP) have been evaluated in the microwave (26–37 GHz) and terahertz (0.2–1 THz) frequency ranges, finding positive results for their use as absorptive materials in electromagnetic (EM) shielding applications for electronic devices [16]. The interesting possibilities of printing electrically conductive structures by using a non-conventional filament made with CNT- and graphene-based polybutylene terephthalate (PBT) are investigated in [17] with due attention also paid to the mechanical stability of the analyzed materials before and after 3D printing. Mechanical properties of 3D-printed polymer specimens are also explored in [18] with a particular focus on identifying the major printing factor that influences them. Even the build orientation in the additive manufacturing process has been considered since it can affect the overall component quality especially in terms of precision and surface finish [19]. Apart from the interest in the electrical and mechanical properties of thermoplastic filaments suitable for the 3D printing, there are potential heat transfer applications of 3D-printed structures. In Reference [20], the effective thermal conductivity of different suitable 3D-printed filaments based on neat polymer and carbon-based polymer composite was measured with an ad-hoc created apparatus after its validation. The effects of the 3D printer parameter setting on the thermal conductivity of filaments made with acrylonitrile butadiene styrene (ABS) are investigated in [21]. First evaluations of the thermal conductivity and mechanical properties for some selected commercial 3D material samples are presented in [22] in order to consider their potential applications in the cryogenic temperature regime.

Although several interesting results have been achieved with an intensive research work, different issues still remain to be solved for printing electrically conductive nanocomposites. In order to really benefit from the advantages due to the combination of AM and nanotechnology, it is necessary to enhance the knowledge of the different available nanomaterials and resultant nanocomposites in terms of general properties and manufacturing processes, as well as identifying the best suited AM techniques to realize the desired structures [23]. Taking advantage of the broad variety of materials requires the development of specific implementations of 3DP. The most relevant ones available in the literature are summarized in [24]. However, there are still uncertainties on the correct design parameters set, manufacturing guidelines, and reproducibility of achieved results. Moreover, further experimental characterizations are needed to enrich the knowledge in this field.

In [25], two different manufacturing processes, i.e., solution blending and melt extrusion, have been compared in terms of rheological and electrical properties of the resulting materials (up to 6 wt % of total charge) based on polylactic acid (PLA) filled with multi-walled carbon nanotubes (MWCNTs), graphene nanoplatelets (GNPs), and a mixture of both fillers (MWCNTs/GNPs). Inferior electrical properties were observed for all composites produced with the solution blending technique. Therefore, in light of these findings, in [26], an electrical characterization in terms of DC conductivity and impedance spectroscopy (magnitude and phase) at low frequency (up to 1 MHz) have been performed for nanocomposites (up to 12 wt % of loading) prepared by melt extrusion. Preliminary results on the thermal conductivity of monophase-composites, filled exclusively with MWCNTs or GNPs, have been reported in [27]. Results on mechanical properties (e.g., nano-indentation and tensile characteristics) have also been discussed [28,29,30].

In the present paper, in order to complete this extensive experimental characterization [25,26,27,28,29,30] and verify the applicability of 3D-manufactured composites in electromagnetic (EM) applications, the EM properties in terms of transmission, reflection, absorption coefficients, and complex permittivity in the frequency range from 26 GHz to 37 GHz (the so-called Ka band) and the thermal conductivities of different formulations are investigated. The behavior of either single-filler (mono-phase) or two-filler (multiphase) composites, differently from the analysis in [27], is explored. The EM and thermal characterization is supported by the morphological investigation achieved by means of high-resolution images. In particular, such analysis allows us to observe the dispersion states of the fillers within the polymer and their mutual interaction, which is important in determining the observed EM and thermal properties. The physical mechanisms linking morphological structures with EM and thermal properties are discussed. The final goal is to apply such nanocomposites in electronics packaging applications requiring either electromagnetic interference (EMI) shielding capability or remarkable thermal properties to dispose the heat produced by the devices. In fact, since the microwave spectrum becomes more and more crowded, the need to electromagnetically shield more components coexisting in the same environment is a current problem that can be solved by introducing innovative materials with suitable properties and new technologies, as additive manufacturing, to produce tailored shapes.

## 2. Materials and Methods

In the present study, the adopted thermosetting polymer is the poly(lactic) acid (PLA) Ingeo™ Biopolymer PLA-3D850 (Nature Works) characterized by fast crystallizing, whereas graphene nanoplates (GNPs) and multiwall carbon nanotubes (MWCNTs) are provided from Times Nano, China. Details on basic properties of fillers and host polymer are summarized in Table 1. PLA has been selected as base thermoplastic polymer in order to favor the development of sustainable and eco-friendly composite materials characterized by good stiffness, strength, and ductility [31]. In [32], electrically conductive nanocomposite filaments for FDM with remarkable electrical and mechanical properties are achieved by using similar fillers but a host polymer, i.e., polyetheretherketone (PEEK) whose recycling is not feasible. In fact, PEEK waste is not biodegradable.

### 2.1. Preparation of Nanocomposites

Three different types of PLA-based nanocomposites were prepared through the melt mixing technique. In particular, nanocomposites of GNP/PLA and MWCNT/PLA with filler concentrations variable from 0 up to 12 wt %, as well as multiphase systems based on GNP/MWCNT/PLA with ratio 50:50 of GNPs to MWCNTs have been produced. Both polymer and nanofillers were dried prior to their use. Firstly, as starting materials, two masterbatches of 12 wt % GNPs and MWCNTs, respectively, were prepared by melt mixing of the filler and the polymer in twin-screw extruder (COLLIN Teach-Line ZK25T) by setting a screw speed of 40 rpm and keeping the temperature in the range 170–180 °C. In fact, they are diluted with neat PLA in a subsequent melt mixing process (masterbatch dilution method) in order to produce mono-filler composite pellets of 1.5%, 3%, 6%, and 9% filler amounts, as well as multiphase composites with equal proportions of both fillers (1.5:1.5, 3:3, and 6:6). After that, the composite pellets were extruded by a single screw extruder (Friend Machinery Co., Zhangjiagang, China) in the temperature range 170–180 °C and a screw speed of 10 rpm for producing filament for 3D printing (FDM) with 1.75 mm in diameter.

### 2.2. 3D Printing (FDM) for Sample Preparation

**Figure 1:**
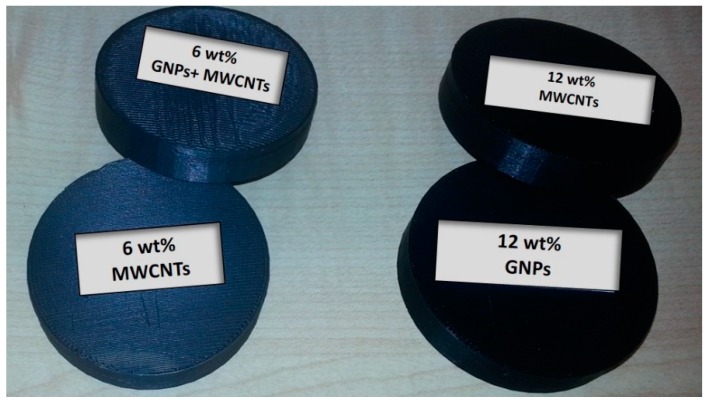
3D-printed disc specimens.

A part of the test samples was prepared by 3D printing (FDM) using the PLA-based nanocomposite filaments, described above in 2.1. Disc specimens (see Figure 1) with a thickness of 10 mm and a diameter of about 50 mm were modeled, and then 3D printed using the 3 types of nanocomposite filaments, GNP/PLA, MWCNT/PLA, and GNP/MWCNT/PLA, with filler contents of 3, 6, 9, and 12 wt %. The fused deposition modeling (FDM-FFF)-type 3D printer X400 PRO German RepRap with an extrusion nozzle with a diameter of 0.5 mm was used. Based on previous experimental tuning, the processing parameters of the 3D printing were a temperature of 200 °C, an extrusion speed of 100 mm/s, and the platform temperature of 60 °C. Samples were printed with a layer height of 0.2 mm and 100% infill, in a rectangular direction of one layer to another.

### 2.3. Experimental Methods 

#### 2.3.1. Scanning Electron Microscopy (SEM) 

**Figure 2:**
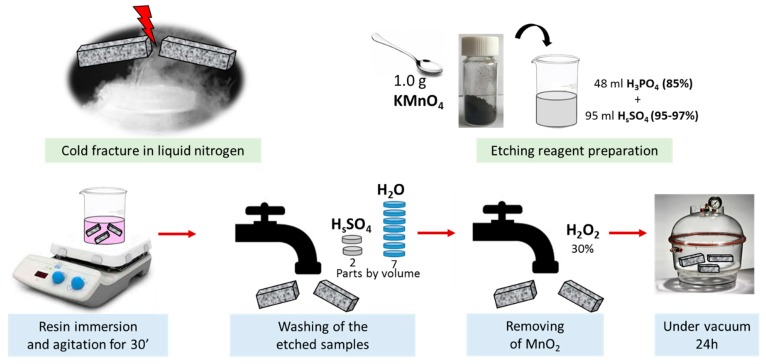
Cold fracture of the samples and etching procedure before the SEM analysis.

Field emission scanning electron microscopy (JSM-6700F, JEOL, Akishima, Japan) operating at 3 kV was used to get information on morphological features of the resulting nanocomposites. Suitable nanocomposites sections were cut with a cold treatment in liquid nitrogen (77 K, −196 °C) and some of them have undergone chemical etching before the observation by SEM, in order to also evaluate the worthiness of this treatment (being classically recommended for the thermosetting resins) for thermoplastic matrices. The etching procedure has been described in detail in [33] and simply schematized in Figure 2.

#### 2.3.2. Thermal Measurements 

**Figure 3:**
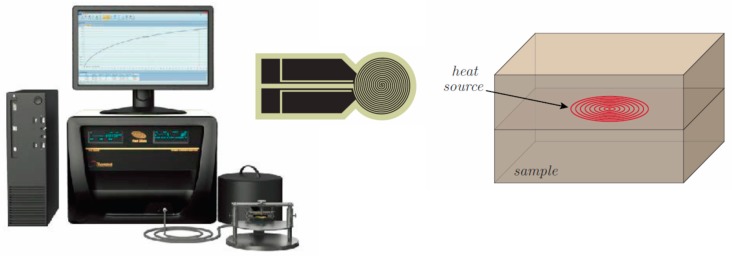
Schematic of the experimental setup for the thermal characterization.

Hot Disk^®^ thermal constants analyzer mod 2500 S (Hot-Disk AB TPS 2500 S, Gothenburg, Sweden) was used to perform thermal conductivity measurements based on the transient plane source technique (TPS) [34,35,36] and according to the ISO 22007-2-2015 standard [37]. More in detail, the TPS element that is placed between two smooth pieces of the sample under test, is an insulator nickel flat disk sensor, provided of concentric and circular line heat sources, which acts simultaneously as heater and temperature sensor (Figure 3). Insulation is guaranteed by a thin layer of kapton, teflon, or mica covering both sides of the probe.

The TPS technique relies on the recording of the resistance changes (against the time) of the heat source serving as the measuring probe [38].

Since the temperature coefficient of the resistance (i.e., *β*) of the sensor is well known (≈4.0 × 10^−3^ K^−1^ at room temperature), its resistance change (i.e., *R*(*t*)) with respect to the initial value *R*_0_ (≈4 Ω at room temperature) before the transient recording allows deriving information on its time-dependent temperature variation ∆*T*(*t*) in agreement with the following relationship:

(1) $$R\left(t\right)=\;R_{0}\left(1+\beta \Delta T\left(t\right)\right).$$

By assuming that the sensor with the features described above is positioned in a sample of infinite dimensions, the thermal properties of the material are evaluated by recording temperature increase over time by means of the formula:

(2) $$\Delta T\left(\tau \right)\;=\frac{P_{0}}{\left(\pi^{3/2}r\lambda \right)}D\left(\tau \right)$$

where the dimensionless time *τ* = (t·α/r^2^)^1/2^ is a function of the measurement time *t*, the thermal diffusivity α, and the radius of the sensor *r*, *P*_0_ is the input heating power, *λ* is the thermal conductivity, and

(3) $$D\left(\tau \right)=\;\frac{1}{{\left[n\left(n+1\right)\right]}^{2}}\int_{0}^{\tau }\sigma^{-2}d\sigma *\left\{\overset{n}{\underset{l=1}{\sum }}l\overset{n}{\underset{k=1}{\sum }}k\;exp\left[\frac{-\left(l^{2}+k^{2}\right)}{4n^{2}\sigma^{2}}\right]I_{0}\left(\frac{lk}{2n^{2}\sigma^{2}}\right)\right\}$$

is a geometric function including the modified Bessel function of the first kind *I*_0_ with *n* representing the number of concentric and equally spaced circular linear sources that make up the probe [39].

On the basis of the above theory, first we must find the value of thermal diffusivity, which actualizes the best linear fit between the temperature increase of the TPS sensor and the geometric function; subsequently, the thermal conductivity is determined by the slope of this straight line, knowing the input heating power *P*_0_ and the sensor radius *r*.

The advantage of the TPS technique relies on the fact that the same TPS element acts as a heat source and a temperature sensor, thus ensuring better accuracy in determining the thermal transport properties compared to other transient-based techniques.

In our case, the measurement of the thermal conductivity was performed on disc-shaped specimens, produced by 3D printing, with a thickness of 10 mm and a diameter of about 50 mm.

A thermal power pulse of magnitude P = 0.1 W for a time measurement of 40 s at room temperature through the sensor (6.40 mm radius *r*) was used. These parameter values ensure that the TPS element is applied to a sample of infinite dimensions. For each sample, five thermal conductivity measurements were performed. The average value is obtained by neglecting the first 50 and the last 10 of 200 points of each recording.

#### 2.3.3. Electrical Measurements 

The DC bulk conductivity of the nanocomposites was measured by using circular-shaped specimens, prepared by hot pressing, of about 50 mm diameter and a thickness of 1 mm. Before carrying out the electrical measurements, the samples are thermally pre-treated at 40 °C for 24 h in order to ensure the evaporation of residual solvents and to avoid effects due to the humidity.

In order to reduce eventual surface roughness and ensure good electrical contacts between the measurement electrode and the specimen, both sides of the latter have been metalized (circular mask of 22 mm of diameter) with silver paint (RS 186-3600 characterized by a volume resistivity 0.001 Ω∙cm). For the DC electrical characterization, performed at room temperature, a multimeter Keithley 6517A configured as both a voltage generator (max ± 1000 V) and a voltmeter (max ± 200 V) and an ammeter HP34401A (min current 0.1 fA) have been adopted. 

To obtain accurate results, five samples for each composition were prepared and then characterized. However, for graphics clarity, only their average values are reported as electrical data.

#### 2.3.4. Electromagnetic Measurements 

**Figure 4:**
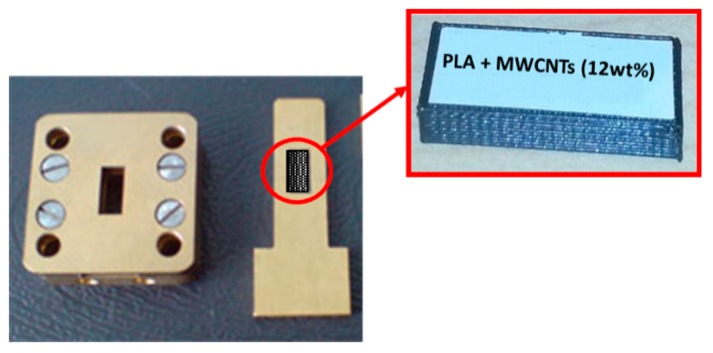
The 7.2 × 3.4 waveguide measurement cell.

The electromagnetic response of the samples is evaluated in terms of scattering parameters. The test samples are films with thickness of 1 mm, prepared by hot pressing. In more detail, transmitted/input (S_21_) and reflected/input (S_11_) signals have been investigated in the so-called Ka-band frequency range (from 26 GHz to 37 GHz) by using a scalar network analyzer R2-408R (ELMIKA, Vilnius, Lithuania) equipped with a 7.2 × 3.4 mm waveguide system. A plane-parallel layer of material was placed in a waveguide cell perpendicular to the incident radiation (Figure 4). They have been mechanically reduced in size in order to fit in the waveguide. Reflection (*R*), transmission (*T*), and absorption (*A*) coefficients are derived from the measured S-parameters as $R=S_{11}^{2}$, $T=S_{21}^{2}\;$, A=1−R−T . The electromagnetic shielding efficiency (EMI) was computed as a sum of absorption and reflection (EMI = *A* + *R*, in %). The complex dielectric permittivity was calculated from the experimental data by the standard method (see Reference [40] for calculation details).

## 3. Results

### 3.1. Morphological Investigation

**Figure 5:**
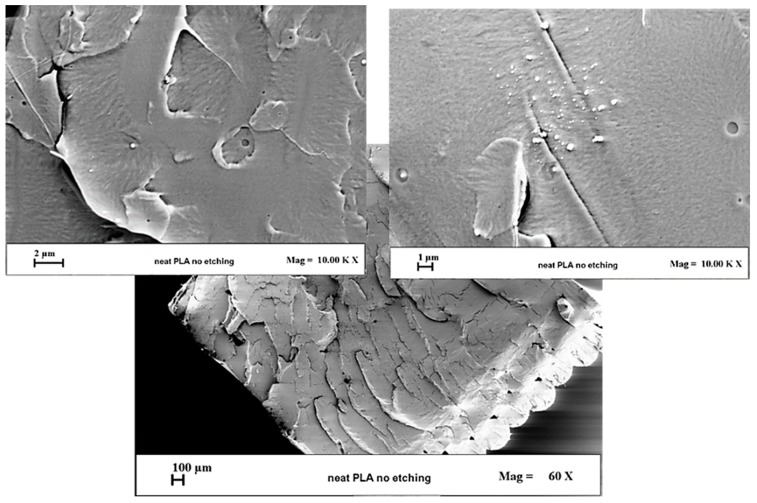
SEM images of pure poly(lactic) acid (PLA) surfaces obtained by cold nitrogen breakage.

**Figure 6:**
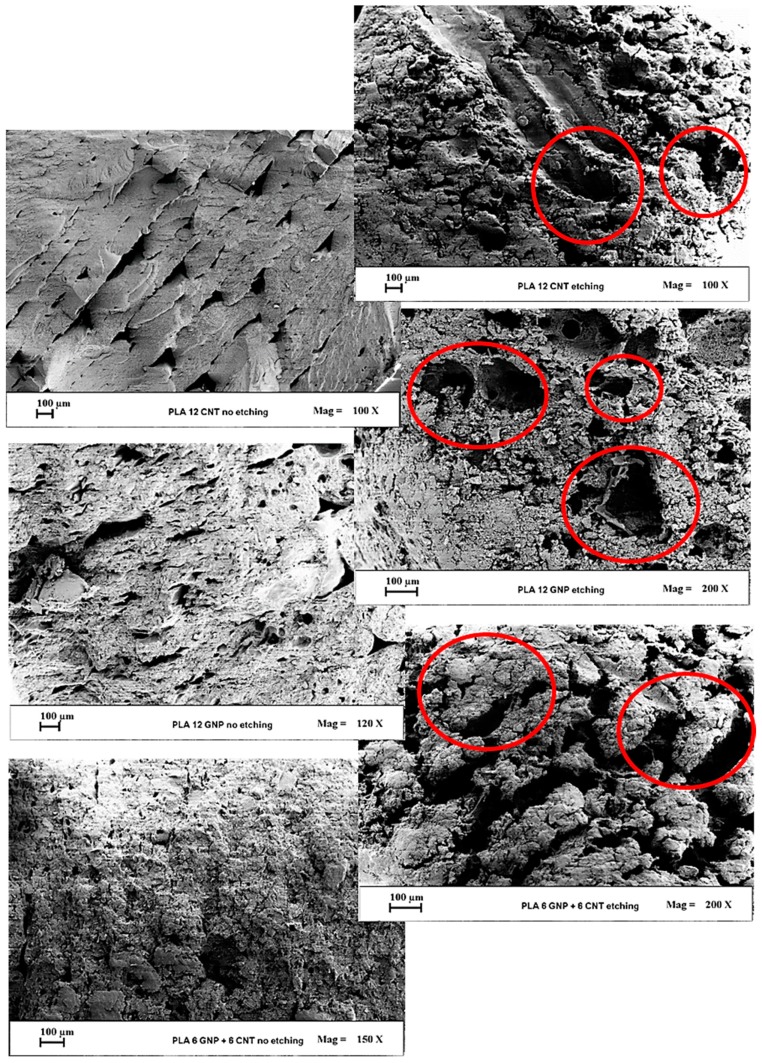
SEM investigation on fracture surfaces of filled composites. First column pictures before etching and the second column after etching.

Scanning electron microscopy (SEM) analysis was performed to get information regarding the dispersion and interactions between the matrix and the adopted fillers. SEM micrographs in Figure 5 concern the surface of pure PLA. Figure 6 (first column) presents the surfaces of composites at 12 wt % filler content obtained by liquid nitrogen breakage carried out prior to examination by SEM. Very different fracture surfaces are visible for the tested PLA and composite materials, which are largely attributed to their brittle or ductile mechanical properties. Microstructurally, the bonded long molecular chains in PLA polymer and interfacial filler–polymer interactions in composites play important roles in the elastic–plastic fracture deformation and crack tip. In Figure 5, the analyzed sections of the neat PLA surface appear very flat due to the ductile fracture type, typical for an isotropic material. In Figure 6 (first column), considerable micro-buckling is developed in the fracture area of the composite surfaces that is typical for more brittle material. The fracture surfaces of GNP/PLA and MWCNT/PLA composites show anisotropic type structure formed by interacted graphene nanoplatelets or carbon nanotubes, as well as voids that cross to both sides of the samples, perpendicular to the loading direction. In contrast, an isotropic structure is developed over the entire surface of the multiphase GNP/MWCNT/PLA sample due, obviously, to the homogeneous network formed by the well-dispersed hybrid fillers and to a fine structure of micro-voids.

Fracture surfaces of the different nanocomposites were etched in accordance with the chemical process schematized in Figure 2 of the previous Section 2.3 Experimental Methods. The reasonableness of this chemical attack for thermoplastic nanocomposites was also investigated through SEM analysis by comparing the results obtained for not etched and etched samples, as shown in Figure 6, first and second column pictures, respectively.

The adoption of etching before carrying out an SEM analysis is highly recommended since the chemical agents are able to remove sufficient parts of the polymer around the fillers, thus creating different sorts of investigable cavities (red circles in Figure 6, second column).

**Figure 7:**
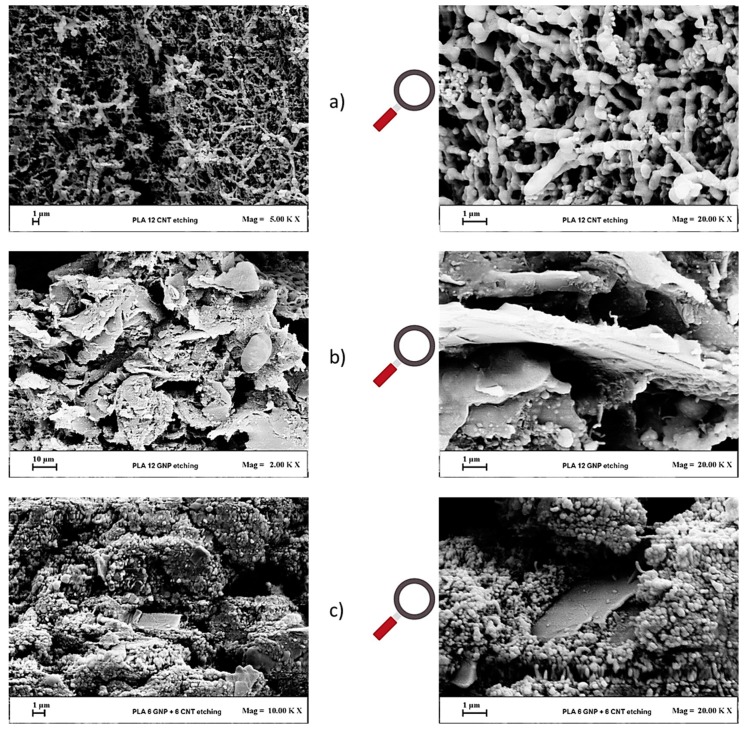
SEM micrographs of nanocomposite at the highest filler concentration, i.e., 12 wt %. In more detail, (**a**) multiwall carbon nanotubes (MWCNTs)-based composites; (**b**) graphene nanoplates (GNPs)-based composites; (**c**) multiphase composites filled with both fillers (MWCNTs + GNPs 1:1). In the left column are low magnifications at 2–10 K and in the right column are their respective magnification at 20 K (1 µm scale).

Therefore, etched samples were chosen hereafter for SEM analysis. Figure 7 shows SEM micrographs of nanocomposite at the highest filler concentration, i.e., 12 wt % of only MWCNTs (Figure 7a), only GNPs (Figure 7b), and a combination of both fillers in equal proportion (Figure 7c). The micrographs in the first column show the etched fracture surfaces in low magnifications 2–10 K and the second column show micrographs at higher magnifications of 20 K.

In all cases and regardless of the type of adopted fillers, their homogeneous dispersion within the PLA and the interconnection of filler particles are observed.

In particular, in Figure 7a, it is possible to observe a well-established continuous percolating network of interconnected nanotubes suitable to promote the electrical conductivity.

In contrast, in Figure 7b it is visible that large, thin, and flat stacks of GNPs with a mean diameter above 10 μm and small thickness are mostly aligned in a direction perpendicular to the fracture load, which is suitable for facilitating conductivity and thermal transfer between the contacted GNPs in the polymer matrix. From Figure 7c, a hybrid structure consisting of a homogeneous network of interconnected MWCNTs, which is interpenetrated by large GNPs, can be observed.

### 3.2. Thermal Conductivity

In recent years, many research efforts have investigated the thermal properties of conductive polymer-based composites for their potential use in the electronic applications. Reliability of service with a concurrent extension of the product life in electronic packaging and energy storage, enhancement in the conversion efficiency of solar energy, rapid heat dissipation in LED devices, and safety issues in batteries are some of the emerging topics where the development of new nanocomposites with improved thermal properties are required. Several materials characterized by remarkable thermal conductivity such as boron nitride, diamond, or carbon-based particles like graphite, nanotubes (CNTs), and graphene (GNPs) have been adopted as reinforcements to improve the thermal transport of polymer nanocomposites. In particular, CNTs and GNPs have attracted great attention for their exceptional intrinsic ability to conduct heat with theoretical values of 3000 (W/mK) and 5000 (W/mK), respectively [41,42]. These nanoparticles, also used concomitantly in a mixture, are considered in this paper in order to obtain enhanced thermal conductivity and strong mechanical stability.

**Figure 8:**
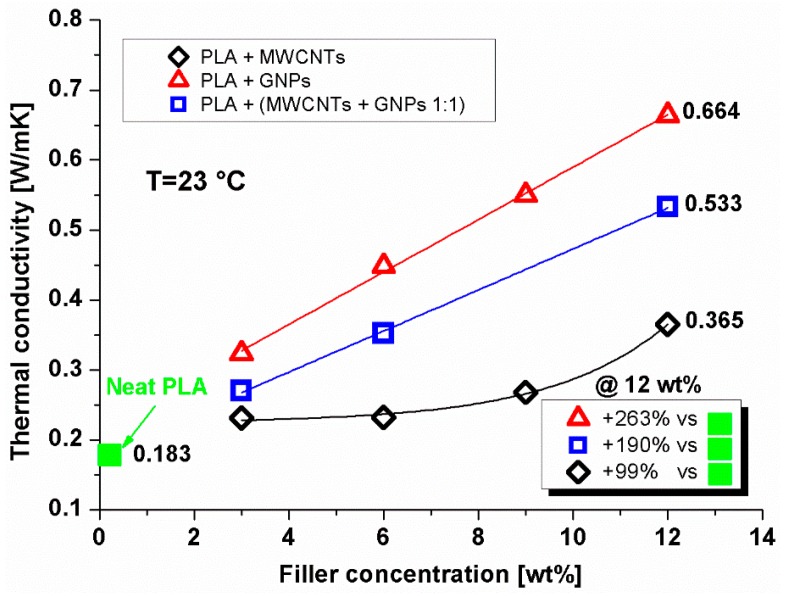
Thermal conductivity of carbon nanotubes (CNT)/PLA, GNP/PLA and (CNT + GNP 1:1)/PLA composites, as varying the filler contents. Markers represent experimental data, whereas lines are the interpolation curves.

Figure 8 shows the thermal conductivity of the different systems obtained by means of the transient plane source analyzer. The achieved results are summarized in Table 2.

It is worth noting that PLA systems containing 2D predominant shape fillers such as graphene nanoparticles (GNPs) showed better heat conduction compared to the other investigated composites. In particular, at the highest GNPs concentration (i.e., 12 wt %) the value of thermal conductivity increased about 263% with respect to the value exhibited by the unfilled resin, whereas an increase of 190% was achieved with the PLA matrix loaded with the same percentage of hybrid nanoparticles (CNT + GNP 1:1 wt %). A smaller but always consistent improvement of about 99% with respect to the neat PLA was observed for systems loaded with carbon nanotubes (CNTs) at 12% in weight.

The evident discrepancies in the thermal conductivity of the investigated nanocomposites can be explained by considering the different mutual interactions established between the filler with the organic polymer. More specifically, the inner surfaces of the twisted 1D dimensional nanotubes were poorly wetted by the PLA and as a consequence, the Kapitza resistance (*Rk*) showed a high value due to the thermal boundary resistance between the two distinct phases (i.e., filler and 3D bulk polymer) characterized by a large difference in the phonon density of states. Differently, when using 2D nanoparticles such as GNPs, their planar shapes improved the interfacial contact area between the reinforcement and the PLA matrix, thus reducing the thermal Kapitza resistance at the interface contacts, compared to what is observed for CNTs. In other terms, the total surface impregnation of the 2D nanoparticles favored the strong binding among the two-dimensional graphene sheets and the PLA thermoplastic matrix. In other terms, the total surface impregnation of the 2D nanoparticles favored the strong binding among the two-dimensional graphene sheets and the PLA thermoplastic matrix, thereby promoting a well-established morphological network of filler within the composite. This means a more effective phononic heat flow since the phonon modes excited in 2D nanoparticles, rather than 1D nanofillers, matched well to those in organic molecules, thus lowering the differences of phonon density of states between the single phases. In the case of combined use of nanoparticles (GNPs + CNTs 1:1 wt %), the measurements carried out show the intermediate value of thermal conductivity in comparison to that found with the use of a single type of nanofiller. The bridge that was established between the two different nanoparticles in the organic matrix (PLA) favored better thermal conduction within the composite material. However, such a property remained lower than that found in the presence of only two-dimensional nanoparticles. The measured thermal conductivity for PLA-based nanocomposites containing variable and hybrid filler contents was comparable to the values reported for previously thermoplastic and epoxy composites containing similar percentages of the same 1D (MWCNTs) and 2D (GNPs) nanoparticles [43,44,45]. In these composites, as it happens for our samples, it was observed that a sufficiently homogeneous dispersion of the same nano-fillers effectively increased the diffusion of phonons and, therefore, the conduction of heat in the material.

### 3.3. Preliminary DC Electrical Properties

**Figure 9:**
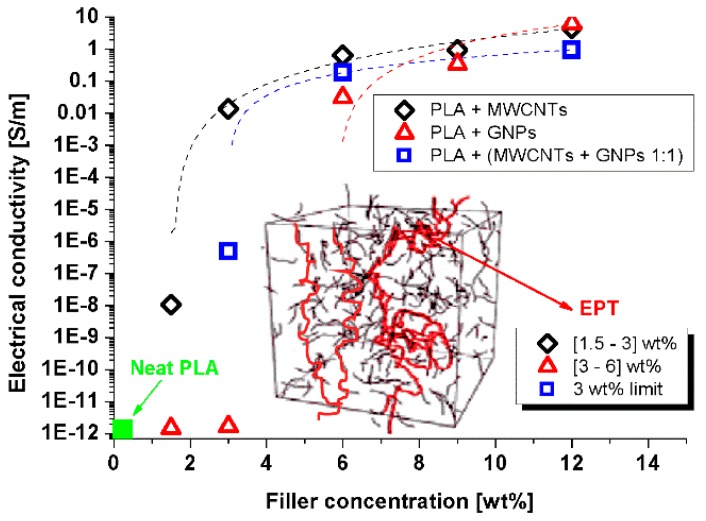
Electrical conductivity versus the filler content (wt %) for the different systems. In the inset, a schematic representation of electric percolation thresholds (EPT) and the values achieved in the present study.

A DC electrical characterization was carried out for obtaining background information such as the electrical conductivity of all the different formulations and the electric percolation thresholds (EPT) for the three different types of composites systems. Figure 9 illustrates the so-called percolation curves. These are obtained by reporting the volume electrical conductivity of the nanocomposites as a function of the fillers loading (wt %). The obtained values are summarized in Table 3. As it was expected, the unfilled polymer showed an electrical conductivity of few pS/m, given its insulating nature. The inclusion of conductive fillers like carbon-based nanomaterials in polymeric matrices is, nowadays, a consolidated method for achieving a transition from the insulating behavior to the conductive state. In fact, an electrical network can be established through the material (the so-called electrical percolation threshold). A small filler amount is required for this purpose, thus preserving the mechanical properties of the polymer along with high electrical conductivity. In agreement with theoretical predictions [46], at low concentrations, the inter-particle separations were above the cut-off tunneling distance (about 1.8 nm, [47]) and the electrical properties were still determined by the host matrix; that is the case of the nanocomposites based on GNPs at 1.5 and 3 wt %, which showed electrical conductivity values strictly close to that of unfilled PLA.

After gradually increasing the loading, fillers began to form a three-dimensional conductive network within the polymer, resulting, beyond the percolation threshold, in a sharp increase of the overall electrical conductivity. The conductivity followed a power-law of the type: $\sigma =\sigma_{0}{\left(\phi -\phi_{C}\right)}^{t}\;$, where *σ*_0_ is the conductivity of the filler, *φ_C_* is the *EPT*, and *t* is a critical exponent linked to the morphological arrangement of the filler in the percolating structure [48]. As evident from Figure 9, at the maximum filler loading (i.e., 12 wt % of total charge) the conductivity showed the value of 4.54 S/m, 6.57 S/m, and 0.95 S/m for nanocomposites prepared with carbon nanotubes, graphene nanoplatelets, and a combination of both fillers, respectively. Regarding the values of the EPT, based on the experimental results, at least an amount of filler ranging within the narrow interval [1.5 ÷ 3.0] wt % a was necessary for obtaining conductive PLA based on CNTs. The EPT lay in the wider [3.0 ÷ 6.0] wt % range for systems with GNPs. For multiphase composites containing both fillers, 3 wt % was a concentration at the limit of the percolation threshold. This noteworthy difference could be explained by accepting that various factors affect the EPT. Among them, the aspect ratio, the specific functionalization, the dispersion state, and the tendency to agglomerate were linked to the filler. As it concerns the matrix, the properties (crystallinity, density, viscosity, etc.) and interaction with the reinforcement have been identified as key factors able to affect the formation of an effective electrically percolating network [49].

The results obtained by our DC experimental characterization are comparable with those reported in a study focused on nonconventional polymer nanocomposites (CNT- and graphene-based polybutylene terephthalate (PBT)) proposed for the printing of electrically conductive structures by means of FDM technology [17]. Instead, if the comparison is performed with those reported in Reference [50], based on the same type of thermoplastic polymer (PLA) and reinforcement (CNTs), an improvement of the DC electrical properties is observed. Most likely, this improvement is due to the different filler aspect ratio and the different physical properties of the polymer (crystallinity, density, viscosity, and so on) since they are produced by different manufacturers. As already mentioned, such parameters are widely recognized as key factors affecting the overall performance of the resulting materials [49].

### 3.4. Electromagnetic Properties in Ka-Band Frequency Range

In the present section, we exclusively consider the polymer composites with the highest filler content (12 wt %) in light of the interesting results regarding the electrical and thermal conductivity observed for such formulations. In particular, their electromagnetic (EM) properties were investigated in the microwave spectrum (26–37 GHz) also referred as Ka-band.

#### 3.4.1. Dielectric Spectroscopy

Electromagnetic properties of the samples are characterized by their relative complex dielectric constant in the considered frequency range, which is commonly expressed as the complex value of:

(4) ε(ω)=ε′(ω)+iε″(ω)

where *ε′* and *ε″* are the real and imaginary parts, respectively, and ω is the angular frequency. More specifically, *ε′* is due to the displacement current and it is influenced by polarization phenomena occurring in the material, whereas *ε″* relates to the losses inside the material [51,52].

**Figure 10:**
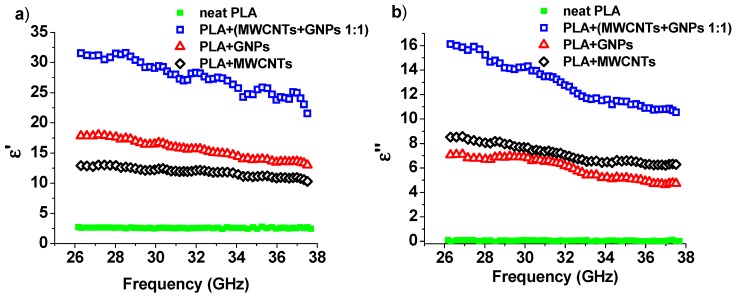
Relative complex permittivity as function of the frequency: Real and imaginary parts in (**a**) and (**b**), respectively.

The measurement temperature was kept constant in this study; hence, its influence on the permittivity behavior can be neglected. Figure 10 shows the frequency dependence of the real part (Figure 10a) and the imaginary part (Figure 10b) of the complex permittivity in the Ka-band (27–37 GHz).

When carbon particles were added into the polymer matrix above the percolation threshold, an increase in both real and imaginary parts of the dielectric permittivity was observed (see Figure 10a,b). In particular, as shown in Figure 10a, the value of real permittivity (at 30 GHz) increased from about 2.6 (neat PLA) up to 12.3, 16.6, and 29.5 for PLA filled with MWCNTs, GNPs, and both fillers, respectively. The imaginary part of the dielectric permittivity for a one-component composite with MWCNTs was slightly higher than the composite with GNPs. The real part behaved in the opposite way. This is due to the aspect ratio for tubes, which was about 5 times greater than that for GNPs. It is proven that the real part of permittivity in nanocomposites materials is related to the polarization capability induced by the nanofiller, mainly along their interfaces between the matrix, i.e., the so-called interfacial polarization [53,54]. Since the particles are involved in the formation of a percolation network in the composite, their contribution to the imaginary part of effective dielectric permittivity *ε″* becomes dominant in comparison to *ε′*, so the decrease in the effective part of the dielectric permittivity for the MWCNTs is understandable.

Electromagnetic properties of the composite (and therefore the dielectric permittivity) depend not only on the type of particles used and their concentrations [55], but mainly on the quality of the distribution of inclusions in the matrix.

To achieve a good dispersion of particles with a huge concentration of them is very difficult, and probably most of the particles in the one-component composite were in the form of agglomerates. The mixture of two fillers improved the dispersion, and as a result, the dielectric permittivity increased significantly compared with single-component composites.

#### 3.4.2. Electromagnetic Properties: Transmission, Reflection, Absorption, and Shielding Efficiency

When an electromagnetic wave impacts on a surface, a part of it is absorbed by the material, another part is reflected, and the rest is transmitted through the material, in agreement with the following power balance:

(5) A+R+T=1

where *A*, *R*, and *T* are the absorption, reflection, and transmission power counterparts, respectively.

In materials showing remarkable electromagnetic shielding features the transmission of the incident electromagnetic field, through the shielding medium, is expected to be ideally zero.

Therefore, the incident field is either absorbed within the shielding material or reflected from it. Otherwise, for no-shielding materials, the transmission value approaches the value one since they are not capable of reflecting or absorbing the incident magnetic field on them.

**Figure 11:**
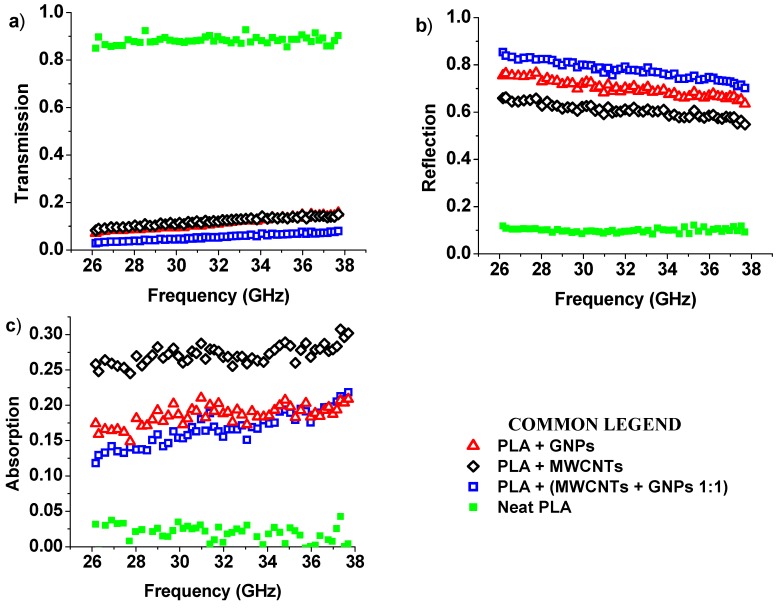
Transmission (**a**), reflection (**b**), and absorption (**c**) spectra in Ka-band for neat PLA and samples at the 12 wt % of total charge.

Figure 11 shows the transmission (Figure 11a), the reflection (Figure 11b), and the absorption (Figure 11c) spectra of the analyzed 0.5 mm thick nanocomposites in the frequency range 26–37 GHz. In fact, it is interesting to note that for neat PLA, the value of *T* was close to 1 in the entire investigated frequency range, thus confirming it to be an EM-transparent material. For it, both the absorption (*A*) and the reflection (*R*) coefficients were close to zero and, as a consequence, an incident electromagnetic field can penetrate with no attenuation (i.e., difference between an electromagnetic signal intensity before and after shielding). This is a typical behavior of plastic materials with almost zero dielectric loss factor (i.e., $tan\delta =\frac{\varepsilon^{\prime \prime }}{\varepsilon^{\prime }}\;$,) which indicates the ability of the material to transform energy into heat) in agreement with the dielectric spectroscopy of the previous Figure 10. Otherwise, the inclusion in the matrix of conductive fillers such as nanotubes and nanoplatelets in concentrations above the percolation threshold can attenuate the penetrating wave level thanks to the effective conductive network established in the material [56]. In this perspective, the best performance was observed for PLA reinforced with GNPs and MWCNTs with a transmission coefficient approaching zero mainly due to its reflection properties (i.e., *R*, about 0.8 at 30 GHz) and partial absorption features (i.e., *A*, about 0.2 at 30 GHz). This confirms the key role of the aspect ratio and the shape of nanofillers in determining the overall electromagnetic performances of the resultant materials as also observed in THz frequency ranges [26]. Table 4 summarizes the values of *T*, *R*, and *A* evaluated at the frequency of 30 GHz. In particular, the electromagnetic shielding efficiency (SE) reported in the last column was calculated as a sum of absorption and reflection (EMI = *A* + *R*, in %). This electromagnetic response is fully consistent with the dielectric constant data presented above. Moreover, the last column of the same table reports, for each investigated formulation, the value of the electromagnetic shielding efficiency (SE), which is an important parameter for efficiently describing the EM features of a medium, depending on the frequency, the distance between the shield and the EM source, the thickness of the shield, and the chemical/physical properties of the material [57].

More in detail, the electromagnetic shielding effectiveness of a material is defined by the ratio of the incident and transmitted field of an electromagnetic wave [57] in accordance with the following mathematical expression:

(6) $$SE\;\left(dB\right)=-20log\left(\left|\frac{E_{t}}{E_{in}}\right|\right)$$

where *E_t_* and *E_in_* are the incident and transmitted field strengths, respectively. The SE provided by Equation (4) can be also expressed ad the sum of three types of different losses encountered by an EM field striking a shield, namely reflection loss (i.e., *R_l_*), absorption loss (i.e., *A_l_*), and multiple reflection loss (i.e., *M_l_*) as follows:

(7) $$SE\;\left(dB\right)=R_{l}\left(dB\right)+A_{l}\left(dB\right)+M_{l}\left(dB\right).$$

The last term *M_l_*, which takes into account additional effects of multiple re-reflections inside the material, is often negligible with respect to *R_l_* and *A_l_* terms, especially in the high frequency range [58] and therefore not considered in the present study. A strong improvement in terms of SE is obtained by harnessing the benefits of the nanotechnology. In fact, the use of carbon-based nanofillers allows us to obtain values for the SE of 10.2, 10.2, and 13.4 dB, respectively, for PLA filled with MWCNTs, GNPs, and both fillers, which are considerably higher than the neat PLA case.

In Reference [59], the authors investigated the electromagnetic interference (EMI) shielding effectiveness of silicon carbide nanofibers (SiCNFs)/epoxy composites in a frequency range (26.5–40 GHz) similar to ours. In particular, they noted for a concentration of 10 wt % of filler, a real part of permittivity (i.e., ε’) of about 9 (at 26.5 GHz) and a SE of 22.9 dB at 40.0 GHz, corresponding to a more than 99% reduction of EMI radiation where absorption was found to be the major shielding mechanism. Instead, in the present paper, at the highest investigated filler concentration (i.e., 12 wt %), for multiphase systems, a three times greater value of ε’ at the same frequency and a comparable SE of 95% was found. The difference could be due to different nature of the resin, thermosetting type in [59] and thermoplastic one in our study, as well as due to the strong difference in the type of adopted filler (carbon nanofibers in [59]), which are key factors influencing the electromagnetic properties and related frequency response of a shielding material.

## 4. Conclusions

Thermal and electromagnetic properties (transmission, reflection, absorption, and shielding efficiency) of a non-conventional thermoplastic polymer based on polylactic acid (PLA, Ingeo™) filled, in different weight percentage, with MWCNTs, graphene nanoplatelets (GNPs), and a combination of both fillers (MWCNTs/GNPs) were investigated. Improved electrical properties in terms of electrical conductivity (up to 4.54 S/m, 6.57 S/m, and 0.95 S/m for the composites prepared with MWCNTs, GNPs, and a combination of both fillers, respectively) along with a significant increase of the thermal properties are achieved, compared with the neat polymer. In fact, composition with 12% of GNPs shows an increase in thermal conductivity of about 263% compared to the unfilled polymer matrix, whereas an improvement of about 99% and 190% is measured for the PLA reinforced with MWCNTs and both fillers, respectively. Regarding the electromagnetic behavior, higher values for the relative complex permittivity are measured for nano-reinforced PLA. Such parameters strongly influence the absorption and attenuation properties of materials and are useful to describe their ability to dissipate energy related to an applied electric field through several polarization mechanisms that commonly result in heat generation. In fact, the shielding efficiency (SE) increases from the low characteristic value of 4% of unfilled polymer up to 95% for multiphase composites containing 12wt % of total charge (MWCNTs + GNPs). These interesting properties are required in order to exploit the potential adoption of such innovative materials for packaging application with electromagnetic interference (EMI) shielding capability. Reflection and absorption features of a shield shall be accompanied by a remarkable thermal conductivity for obtaining good dissipation of the energy by Joule effect. Further experimental characterization combined with a theoretical study is needed to identify the best combination of the two adopted fillers. In more detail, by means of robust design (RD) approach, the overall optimization of the material properties and production process can be searched for also in view of the cost reduction [60].

## Figures and Tables

**Table 1:** Features of the adopted host polymer and carbon-based fillers.

Property	Phase		
	Polymer	GNP	MWCNT-OH
Commercial code	PLA-3D850	TNIGNP	TNIMH4
Purity (wt %)	x	90	95
Thickness (nm)	x	<30	x
Peak melt temperature (°C)	165–180	x	x
Glass transition Temp. (°C)	55–60	x	x
MFR, g/10 min *	7–9	x	x
Average size (µm)	x	5–7	x
External diameter, (nm)	x	x	10–30
Length (µm)	x	x	10–30
OH-content (%)	x	x	2.48
Aspect ratio	x	~240	~1000
Density (g/cm^3^)	x	2.2	2.1

* 210 °C/2.16 kg—D1238 ASTM.

**Table 2:** Thermal conductivity (W/mK).

Filler (wt %)	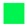 Neat PLA	Δ PLA + GNPs	◊ PLA + MWCNTs	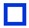 PLA + (MWCNTs + GNPs 1:1)
0	0.183	x	x	x
3.0	x	0.323	0.231	0.270
6.0	x	0.448	0.232	0.352
9.0	x	0.550	0.268	x
12.0	x	0.664	0.365	0.533

**Table 3:** DC electrical conductivity (S/m).

Filler (wt %)	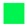 Neat PLA	Δ PLA+GNPs	◊ PLA+MWCNTs	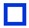 PLA+(MWCNTs+GNPs 1:1)
0	1 × 10^−12^	x	x	x
1.5	x	1.50 × 10^−12^	1.08 × 10^−8^	x
3.0	x	1.70 × 10^−12^	1.40 × 10^−2^	5.02 × 10^−7^
6.0	x	3.12 × 10^−2^	6.57 × 10^−1^	1.85 × 10^−1^
9.0	x	3.47 × 10^−1^	9.40 × 10^−1^	x
12.0	x	6.27	4.54	9.50 × 10^−1^

**Table 4:** Electromagnetic properties at 30 GHz.

Composite	T	R	A	SE (dB)	SE (%)
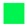 Neat PLA	0.96	0.02	0.02	0.20	4
Δ PLA+GNPs	0.09	0.71	0.19	10.22	89
**◊** PLA+MWCNTs	0.09	0.71	0.19	10.22	89
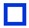 PLA+(MWCNTs+GNPs 1:1)	0.05	0.79	0.16	13.45	95

## References

[1] Xin W., Man J., Zuowan Z., Jihu G., David H. (2017). 3D printing of polymer matrix composites: A review and prospective. Compos. B Eng..

[2] Ngo T.D., Kashani A., Imbalzano G., Nguyen K.T.Q., Hui D. (2018). Additive manufacturing (3D printing): A review of materials, methods, applications and challenges. Compos. B Eng..

[3] Kruth J.P., Leu M.C., Nakagawa T. (1998). Progress in additive manufacturing and rapid prototyping. CIRP Ann..

[4] Wong K.V., Hernandez A. (2012). A review of additive manufacturing. ISRN Mech. Eng..

[5] Surange V.G., Punit V.G. (2016). 3D printing process using fused deposition modelling (FDM). Int. J. Res. Eng. Technol. (IRJET).

[6] Tofail S.A., Koumoulos E.P., Bandyopadhyay A., Bose S., O’Donoghue L., Charitidis C. (2018). Additive manufacturing: Scientific and technological challenges, market uptake and opportunities. Mater. Today.

[7] Ivanova O., Williams C., Campbell T. (2013). Additive manufacturing (AM) and nanotechnology: Promises and challenges. Rapid Prototyp. J..

[8] Rouhollah D., Farahani R.D., Dubé M., Therriault D. (2016). Three-Dimensional Printing of Multifunctional Nanocomposites: Manufacturing Techniques and Applications. Adv. Mater..

[9] Angjellari M., Tamburri E., Montaina L., Natali M., Passeri D., Rossi M., Terranova M.L. (2017). Beyond the concepts of nanocomposite and 3D printing: PVA and nanodiamonds for layer-by-layer additive manufacturing. Mater. Des..

[10] Viskadourakis Z., Vasilopoulos K.C., Economou E.N., Soukoulis C.M., Kenanakis G. (2017). Electromagnetic shielding effectiveness of 3D printed polymer composites. Appl. Phys. A.

[11] Adams J.J., Slimmer S.C., Lewis J.A., Bernhard J.T. (2015). 3D-printed spherical dipole antenna integrated on small RF node. Electron. Lett..

[12] Mirzaee M., Noghanian S., Chang Y. (2017). Low-pro le bowtie antenna with 3D printed substrate. Microw. Opt. Technol. Lett..

[13] Kim O.S. (2013). 3D printing electrically small spherical antennas. 2013 IEEE Antennas and Propagation Society International Symposium (APSURSI).

[14] Ahn B.Y., Duoss E.B., Motala M.J., Guo X., Park S.I., Xiong Y., Yoon J., Nuzzo R.G., Rogers J.A., Lewis J.A. (2017). Omnidirectional Printing of Flexible, Stretchable, and Spanning Silver Microelectrodes. Microw. Opt. Technol. Lett..

[15] Kestilä A., Nordling K., Miikkulainen V., Kaipio M., Tikka T., Salmi M., Auer A., Leskelä M., Ritala M. (2018). Towards space-grade 3D-printed, ALD-coated small satellite propulsion components for fluidics. Addit. Manuf..

[16] Bychanok D., Angelova P., Paddubskaya A., Meisak D., Shashkova L., Demidenko M., Plyushch A., Ivanov E., Krastev R., Kotsilkova R. (2018). Terahertz absorption in graphite nanoplatelets/polylactic acid composites. J. Phys. D Appl. Phys..

[17] Gnanasekaran K., Heijmans T., van Bennekom S., Woldhuis H., Wijnia S., de With G., Friedrich H. (2017). 3D printing of CNT- and graphene-based conductive polymer nanocomposites by fused deposition modeling. Appl. Mater. Today.

[18] Sagias V.D., Giannakopoulos K.I., Stergiou C. (2018). Mechanical properties of 3D printed polymer specimens. Procedia Struct. Integr..

[19] Taufik M., Jain P.K. (2013). Role of build orientation in layered manufacturing: A review. Int. J. Manuf. Technol. Manag..

[20] Flaata T., Michna G.J., Letcher T. Thermal conductivity testing apparatus for 3D printed materials. Proceedings of the ASME 2017 Summer Heat Transfer Conference.

[21] Sonsalla T., Moore A.L., Meng W.J., Radadia A.D., Weiss L. (2018). 3-D printer settings effects on the thermal conductivity of acrylonitrile butadiene styrene (ABS). Polym. Test..

[22] Weiss K.-P., Bagrets N., Lange C., Goldacker W., Wohlgemuth J. (2015). Thermal and mechanical properties of selected 3D printed thermoplastics in the cryogenic temperature regime. IOP Conf. Ser. Mater. Sci. Eng..

[23] Gao W., Zhang Y., Ramanujan D., Ramani K., Yong Chen Y., Williams C.B., Wange C.L., Shin Y.C., Zhang S., Zavattieri P.D. (2015). The status, challenges, and future of additive manufacturing in engineering. Comput. Aided Des..

[24] Utela B., Storti D., Anderson R., Ganter M. (2008). A review of process development steps for new material systems in three dimensional printing (3DP). J Manuf Process.

[25] Spinelli G., Lamberti P., Tucci V., Kotsilkova R., Tabakova S., Ivanova R., Angelova P., Angelov V., Ivanov E., Di Maio R. (2018). Morphological, Rheological and Electromagnetic Properties of Nanocarbon/Poly(lactic) Acid for 3D Printing: Solution Blending vs. Melt Mixing. Materials.

[26] Spinelli G., Lamberti P., Tucci V., Ivanova R., Tabakova S., Ivanov E., Kotsilkova R., Cimmino S., Di Maio R., Silvestre C. (2019). Rheological and electrical behaviour of nanocarbon/poly(lactic) acid for 3D printing applications. Compos. B Eng..

[27] Lamberti P., Spinelli G., Kuzhir P., Guadagno L., Naddeo C., Romano V., Kotsilkova R., Angelova P., Georgiev V. (2018). Evaluation of Thermal and Electrical Conductivity of Carbon-based PLA Nanocomposites for 3D Printing. AIP Conf. Proc..

[28] Kotsilkova R., Petrova-Doycheva I., Menseidov D., Ivanov E., Paddubskaya A., Kuzhir P. (2019). Exploring thermal annealing and graphene-carbon nanotube additives to enhance crystallinity, thermal, electrical and tensile properties of aged poly(lactic) acid-based filament for 3D printing. Compos. Sci. Tech..

[29] Batakliev T., Petrova-Doycheva I., Angelov V., Georgiev V., Ivanov E., Kotsilkova R., Casa M., Cirillo C., Adami R., Sarno M. (2019). Effects of Graphene Nanoplatelets and Multiwall Carbon Nanotubes on the Structure and Mechanical Properties of Poly(lactic acid) Composites: A Comparative Study. Appl. Sci..

[30] Batakliev T., Georgiev V., Ivanov E., Kotsilkova R., Di Maio R., Silvestre C., Cimmino S. (2018). Nanoindentation analysis of 3D printed poly(lactic acid)-based composites reinforced with graphene and multiwall carbon nanotubes. J. Appl. Polym. Sci..

[31] Stoof D., Pickering K. (2018). Sustainable composite fused deposition modelling filament using recycled pre-consumer polypropylene. Compos. B Eng..

[32] Gonçalves J., Lima P., Krause B., Pötschke P., Lafont U., Gomes J.R., Abreu C.S., Paiva M.C., Covas J.A. (2018). Electrically conductive polyetheretherketone nanocomposite filaments: From production to fused deposition modeling. Polymers.

[33] Guadagno L., Raimondo M., Vertuccio L., Mauro M., Guerra G., Lafdi K., De Vivo B., Lamberti P., Spinelli G., Tucci V. (2015). Optimization of graphene-based materials outperforming host matrices. RSC Adv.

[34] Gustafsson S.E. (1991). Transient plane source techniques for thermal conductivity and thermal diffusivity measurements of solid materials. Rev. Sci. Instrum..

[35] Ghosh S., Teweldebrhan D., Morales J.R., Garay J.E., Balandin A.A. (2009). Thermal properties of the optically transparent pore-free nanostructured yttria-stabilized zirconia. J. Appl. Phys..

[36] Romano V., Naddeo C., Vertuccio L., Lafdi K., Guadagno L. (2017). Experimental Evaluation and Modelling of Thermal Conductivity of Tetrafunctional Epoxy Resin Containing Different Carbon Nanostructures. Pol. Eng. Sci..

[37] (2015). (ISO 22007-2:2015), Plastics—Determination of Thermal Conductivity and Thermal Diffusivity—Part 2: Transient Plane Heat Source (Hot Disc) Method.

[38] Dupleix A., Kusiak A., Hughes M., Rossi F. (2012). Measuring the thermal properties of green wood by the transient plane source (TPS) technique. Holzforschung.

[39] Zhang H., Li T.-M., Tao W.-Q. (2017). Theoretical accuracy of anisotropic thermal conductivity determined by transient plane source method. Int. J. Heat Mass Transf..

[40] Bychanok D., Plyushch A., Piasotski K., Paddubskaya A., Voronovich S., Kuzhir P., Baturkin S., Klochkov A., Korovin E., Letellier M. (2015). Electromagnetic properties of polyurethane template-based carbon foams in Ka-band. Phys. Scr..

[41] Hone J., Whitney M., Piskoti C., Zettl A. (1999). Thermal conductivity of single-walled carbon nanotubes. Phys. Rev. B.

[42] Balandin A.A. (2001). Thermal properties of graphene and nanostructured carbon materials. Nat. Mater..

[43] Noh Y.J., Kim H.K., Ku B.-C., Khil M.-S., Kim S.Y. (2016). Thermal conductivity of polymer composites with geometric characteristics of carbon allotropes. Adv. Eng. Mat..

[44] Kim S.H., Bae H.S., Yu J., Kim S.Y. (2016). Thermal conductivity of polymer composites with geometrical characteristics of graphene nanoplatelets. Sci. Rep..

[45] Yan H., Tang Y., Lang W., Li Y. (2014). Enhanced thermal conductivity in polymer composites with aligned graphene nanosheets. J. Mat. Sci..

[46] Stauffer D., Aharony A. (2003). Introduction to Percolation Theory.

[47] De Vivo B., Lamberti P., Spinelli G., Tucci V. (2014). A morphological and structural approach to evaluate the electromagnetic performances of composites based on random networks of carbon nanotubes. J. Appl. Phys..

[48] Nan C.-W., Shen Y., Ma J. (2010). Physical properties of composites near percolation. Annu. Rev. Mater. Res..

[49] De Vivo B., Lamberti P., Spinelli G., Tucci V., Guadagno L., Raimondo M. (2015). The effect of filler aspect ratio on the electromagnetic properties of carbon-nanofibers reinforced composites. J. Appl. Phys..

[50] Urquijo J., Dadreou S., Echevarria G., Eguiazabal J.I. (2017). Morphology and properties of electrically and rheologically percolated PLA/PCL/CNT nanocompoistes. J. Appl. Polym. SCi..

[51] Tamura R., Lim E., Manaka T., Iwamoto M. (2006). Analysis of pentacene field effect transistor as a Maxwell-Wagner effect element. J. Appl. Phys..

[52] Arjmand M., Mahmoodi M., Park S., Sundarara U.T. (2013). An innovative method to reduce the energy loss of conductive filler/polymer composites for charge storage applications. Compos. Sci. Technol..

[53] Panwar V., Park J.O., Park S.H., Kumar S., Mehra R.M. (2010). Electrical, dielectric, and electromagnetic shielding properties of polypropylene-graphite composites. J. Appl. Polym. Sci..

[54] Yuan J.-K., Yao S.-H., Dang Z.-M., Sylvestre A., Genestoux M., Bai J. (2011). Giant Dielectric Permittivity Nanocomposites: Realizing True Potential of Pristine Carbon Nanotubes in Polyvinylidene Fluoride Matrix through an Enhanced Interfacial Interaction. J. Phys. Chem. C.

[55] Ameli A., Wang S., Kazemi Y., Park C.B., Pötschke P. (2015). A facile method to increase the charge storage capability of polymer nanocomposites. Nano Energy.

[56] Arjmand M., Mahmoodi M., Gelves G.A., Park S., Sundararaj U. (2011). Electrical and electromagnetic interference shielding properties of flow-induced oriented carbon nanotubes in polycarbonate. Carbon.

[57] Clayton R.P. (2006). Introduction to Electromagnetic Compatibility.

[58] Celozzi S., Araneo R., Lovatr G., Clayton P. (2008). Electromagnetic Shielding.

[59] Zhang Y., Wang Z., Zhang B., Zhao G.-L., Guo S.M. (2015). The electromagnetic interference shielding effectiveness of high aspect-ratio SiC nanofibers/epoxy composites. RSC Adv..

[60] De Vivo B., Lamberti P., Spinelli G., Tucci V., Guadagno L., Raimondo M., Vertuccio L., Vittoria V. (2013). Improvement of the electrical conductivity in multiphase epoxy-based MWCNT nanocomposites by means of an optimized clay content. Comp. Sci. Technol..

